# Chats about Medical Museums

**Published:** 1887-05-28

**Authors:** 


### Chats About Medical Museums.

By One of the Crowd.

(Continued from p. 125, vol. ii.)

VI.—St. Bartholomew's Hospital.

If there is anything in the medical museums of London more amazingly intricate and puzzling than the models displaying the interior mechanism of the human body, it must be some of the catalogues that are mixed up with the models for the enlightenment of the ignorant. This is especially the case at St. Bartholomew's, where much of the interest of a large miscellaneous collection is entirely lost to the uninitiated for want of an intelligible catalogue, or what would be better still, explanatory labels attached to the objects. There is a catalogue, but it is in several separate parts; and if you want to refer to any particular number, you are sure to find that the only parts at hand do not contain that number, and that if you must have the proper part, you may have to set out on a voyage of discovery all round the museum. The case is pretty much the same in most of the other museums. To the unprofessional outsider this very seriously detracts from the interest of these institutions; but then, no doubt, it may be said that they are not intended to interest the unprofessional outsider. They are for scientific purposes only, and if from the Cimmerian darkness of the outer world the uninstructed will steal in to these centres of light, it is mere presumption on their part to complain that the illumination is not as perfect as it might be.

The museum at St. Bartholomew's is very similar to that at St. Thomas's, already referred to. It is a large and lofty building, lighted from the roof. Outside staircases give access to two galleries, one above the other, both ground-floor and galleries being pretty well filled with anatomical models, pathological preparations, objects illustrative of comparative anatomy, surgical instruments, cases filled with specimens of drugs, and so forth. There are two or three skeletons of men of gigantic stature, and several of tiny dimensions, especially some presenting remarkable instances of water on the brain—hydrocephalus—by which the heads are expanded to enormous dimensions. In one diminutive skeleton the face is reduced comparatively to quite insignificant proportions—a mere appendage to the upper portion of the skull, which is twenty inches in circumference and eight in its longest diameter, though the disease was congenital, and the little creature who owned the puny framework cannot have lived long enough to grow much beyond the proportions of its birth.

By the way, there are here preserved in spirit several freaks of nature, in the shape of two-headed babies, and so forth, as indeed there are in the other museums we have noticed; only, perhaps it is as well not to say too much about curiosities of this kind, or The Hospital may by-and-bye be held responsible for more of them. Notwithstanding that science has, to a very great extent, exploded the idea that mental impressions can have anything to do with these matters, there are many ladies who tenaciously hold on to their right to make themselves miserable with it. Here they are, however, several of them, the queerest little objects imaginable, and side by side, too, with embryo elephants and fœtal kangaroos. At St. Thomas's they have one little creature, which undoubtedly would have been another specimen of "the pig-faced lady," once so notorious as an exhibition in this country, only that, fortunately, nature, having made a hideous mistake, declined to proceed further in the matter, and life was mercifully arrested at the outset.

Among the things likely to arrest the attention of the casual visitor to St. Bartholomew's Museum is the mummy of a cat, possibly the object of worshipful veneration by some Egyptian or other at the time the stone-masons of Heliopolis were carving the hieroglyphics on our Thames Embankment obelisk,—if it is really a genuine mummy, and not the antique outcome of the wit of some modern wag. Near the cat is the undoubted mummy of a child, buried in 1777, and, it is to be presumed, kept in a singular state of preservation by something or other in the soil from which its grave was dug. Another object worth a passing notice is one of the blind fishes of the Kentucky Caves. Most persons, it may be presumed, are aware that in the dark, subterranean lakes of those famous caves there are fish which, through countless generations, have found no use for eyesight, and have either never developed the visual organ, or, having once possessed it, have lost it from mere disuse. At all events they are sightless.

On the shelves in the galleries too are some curious specimens of what may at first sight be taken to be examples of what may be done by some manufacturer's patent marking-ink. They are, in fact, pieces of human skin elaborately tattooed, taken from the bodies of "subjects" in the dissecting-room. It is really very curious to observe what a singular *penchant* certain sections of the community have for permanently adorning their own skins. Is it a survival of an aboriginal instinct, transmitted from the early Britons? Hospital authorities know how common it is, and so also do jail authorities, and those who are personally engaged in the work of selecting recruits for the army and navy. Every recruit has to undergo bodily examination, and any indelible mark anywhere about his skin is recorded in a book, as a means of future identification if it should be needed. The same is the case with persons convicted of crime, and these records are here and there very curious. The same would no doubt be the case with hospital patients if similar records were kept. This, of course, is not done, but most medical museums have one or two of the most remarkable examples of the kind of thing met with in the course of *post-mortem* examinations. Talking of skins reminds one of a gigantic model in one of the cases in this museum, standing boldly up without a skin, and looking with his blue veins and red arteries very much like a backwoods Indian in his war-paint. The models in this museum, though, are not particularly striking. Such as there are, are for the most part in plaster, white or coloured, and, after the wax anatomical and pathological models in Guy's, are very artificial-looking affairs.

But though not very rich in models, St. Bartholomew's has a fine array of natural preparations dispersed in glass cases round the galleries. One very striking and impressive feature recently added to the ground-floor of the institution is a series of exceedingly skilful coloured drawings of diseases of the tongue, most of them being representations of actual cases treated in the hospital. The maladies are many of them very shocking, and, as may be readily supposed, they some of them throw rather a ghastly light on some phases of "life." But the most remarkable object on the ground-floor of this museum is not of this class. All such places have their collections of calculi taken from the human system; but probably no other museum in the world has just such a collection of them as are exhibited in one small glass case. There are a number of small ones of various sizes, and two large ones. Altogether they weigh 47 oz.—nearly three pounds of stone—and they were all the accretions of one pair of kidneys!

(To be continued.)

